# Meal plans for meeting the reference values using food items available in shelters

**DOI:** 10.1186/s40795-023-00726-9

**Published:** 2023-06-24

**Authors:** Tamaki Takeda, Noriko Sudo, Nobuyo Tsuboyama-Kasaoka, Ikuko Shimada, Keiichi Sato, Yuki Shibamura, Sayaka Nagao-Sato

**Affiliations:** 1 The Graduate School of Humanities and Sciences, Ochanomizu University, Tokyo, Japan; 2 Natural Science Division, Faculty of Core Research, Ochanomizu University, Tokyo, Japan; 3 Section of Global Disaster Nutrition, International Center for Nutrition and Information, National Institute of Health and Nutrition, National Institutes of Biomedical Innovation, Health and Nutrition, Osaka, Japan; 4 Department of Nutrition, Faculty of Nutrition, University of Kochi, Kochi, Japan; 5 School of Network and Information, Senshu University, Kawasaki, Japan

**Keywords:** Evacuation shelter, Food aid, Nutrition assistance, Meal plan, Nutritional profile, Natural disaster

## Abstract

**Background:**

We have suggested “Revised Nutritional Reference Values for Feeding at Evacuation Shelters” (Revised RV) as a daily nutritional recommendation for meals served at evacuation shelters where poor diets had been reported. Since there are no meal examples to satisfy the Revised RV, our objectives were, for the future meal provision, to develop nutritionally adequate meal plans using the foods served at shelters in the past and to examine if the Revised RV could be met by changing combination of foods available.

**Methods:**

In this case study using secondary data, we analyzed food weights of 86 meals served and recorded at 12 shelters after the heavy rains in July 2020. We obtained these data from Kumamoto Prefecture that was damaged and asked us dietary assessment for nutrition assistance. Foods were classified into 3 types according to the check mark in the record sheets: food aid (commercial packaged food), boxed meal, and hot meal service. We counted serving frequency of each food and analyzed nutritional differences by their combinations. Menus were devised by choosing foods that were served more frequently or were more nutritious among those served at shelters. The target values for one meal were set at 1/3 of the Revised RV for energy, protein, vitamins B_1_, B_2_, and C, and salt.

**Results:**

None of the meals served in the shelters satisfied the target. We created 2 menus using food aid only: (#1 curry doughnut, milk with long shelf-life, and orange jelly) and (#2 salmon rice ball, ham and cheese sandwich, and vegetable juice); 1 menu by combination of boxed meal and food aid: (#3 boxed meal and vegetable juice); and 2 menus by combination of hot meal service and food aid: (#4 chicken meatball soup, packaged tofu, soy sauce, preprocessed white rice, and bottled green tea) and (#5 bamboo shoots rice, chicken and vegetable miso soup, and bottled green tea). Planned menus generally contained more energy, protein, and vitamins and less salt than the meals served. Their vitamin C contents were especially higher.

**Conclusion:**

Nutritionally adequate meals could be planned by changing the combination of foods available in shelters.

**Supplementary Information:**

The online version contains supplementary material available at 10.1186/s40795-023-00726-9.

## Background

In the event of a major disaster, many affected residents will be forced to live in evacuation shelters. Nutritional support is necessary to maintain the health of disaster victims. The National Institute of Health and Nutrition and the Japan Dietetic Association established four post-disaster phases according to the time after the disaster and indicated nutrients that should be considered in each post-disaster phase; Phase 0 (the day of disaster) and 1 (within 72 h after the disaster): energy; Phase 2 (four days to one month after the disaster) and 3 (beyond one month after the disaster): energy, protein, vitamins, and minerals [1]. However, in disaster areas in the past, food provided in evacuation shelters did not meet the nutrition needs of evacuees. According to 24-h dietary recalls in two shelters during the 1999 Greek Earthquake, inadequate intakes of energy and protein were observed among adults, especially the elderly [2]. In the Great East Japan Earthquake, an oversupply of carbohydrate-rich foods and a shortage of dairy products, meat, fish, and vegetables were reported in shelters 1 month after the earthquake [3]. World Health Organization (WHO) and previous studies have indicated that unbalanced diets after natural disasters may have negative impacts on mental and physical health [4–6].

As a standard for nutrition in emergencies, the WHO and the Sphere Association use 2,100 kcal/person/day for energy intake, also provide recommendations for daily intake of micronutrients and the proportion of protein and fat [4, 7]. In Japan, the Ministry of Health, Labour and Welfare (MHLW) issued the Nutritional Reference Values for dietary planning at emergency shelters (RV), i.e., the target daily intakes per person for energy, protein, and vitamins B_1_, B_2_, and C [8]. These nutrients are considered a top priority in a nutritional plan as they will become deficient at an early stage after a natural disaster [9]. The three micronutrients were selected since they have a short storage period in the body and their deficiencies were common in shelters. On the other hand, some minerals like calcium and iron have low priority within three months after disaster, and therefore, their values were not set for this period. In 2021, the RV was modified as “Revised Nutritional Reference Values for Feeding at Evacuation Shelters” (Revised RV) [10] based on the latest Dietary Reference Intakes for Japanese 2020. The Revised RV was 2000 kcal for energy, 55 g for protein, 0.9 mg for vitamin B_1_, 1.0 mg for vitamin B_2_, 80 mg for vitamin C, and < 8.0 g for salt per person per day. The Revised RV for salt was set to prevent hypertension, according to the goal in Health Japan 21 (the second term), Japan’s latest national health promotion plan [11]. In shelters, preparing a menu that satisfies nutritional recommendations in advance is necessary to ensure that meals can be promptly provided with nutritional considerations [12]. However, few meal examples that met these nutrition recommendations were suggested [9, 13]; furthermore, there is no indication regarding the specific meal examples to satisfy the Revised RV. Additionally, food availability after a disaster is limited, and the menus designed for non-emergency situations may not be directly applicable in times of disaster. Establishing the menu based on the foods provided at evacuation shelters in the past would be highly feasible, even in times of disaster.

For nearly a month from July third, 2020, a wide area from western to eastern Japan experienced heavy rains. Some municipalities in Kumamoto Prefecture, one of the most affected areas, opened emergency shelters and provided the evacuees with food and water. In the present study, we reported the contents of food and meals served in the shelters. Since there are no meal examples to satisfy the Revised RV, our objectives were, for the future meal provision, to develop nutritionally adequate meal plans using the foods served at shelters in this disaster and to examine if the Revised RV could be met by changing combination of foods available.

## Methods

### Study design and settings

**Fig. 1:**
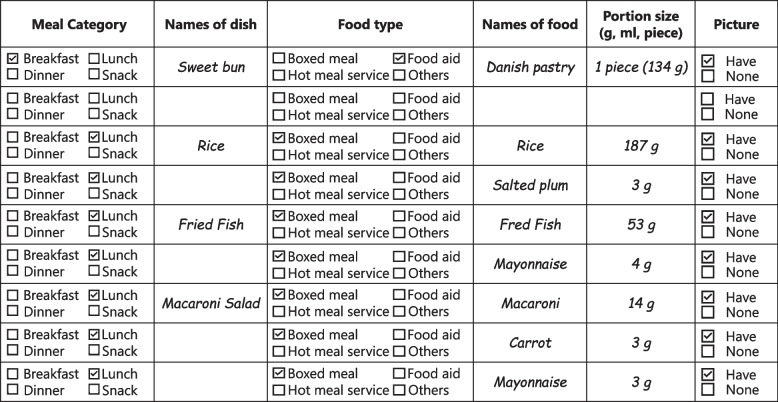
Excerpt of record sheet. Italics and checks are examples

This was a case study with secondary analysis. Weight measurement of foods served at shelters were conducted from July 19 to 23 (16–20 days after the disaster) by the prefectural government as a part of public services to improve the situation of shelters. The prefectural government collected dietary data from 12 shelters with relatively large numbers of evacuees (15–300 people per shelter) in the six affected municipalities. Shelters with more evacuees had reportedly low nutrient supply and less frequent meal provision [3]. At the time of the survey, meals were prepared in the shelters by evacuees and/or aid organizations, or the shelters received ready-to-serve items including food aids and boxed meals. Administrative dietitians and/or volunteer dietitians dispatched from dietetic associations were requested by the prefectural government to collect data of meals served at shelters. They followed the written instruction provided by the authors and used the record sheet (Fig. 1) and dietary assessment sheet for evacuation shelters (Supplementary material) [14]. Both sheets were developed by the authors and officially used by the Japan Dietetic Association-Disaster Assistance Team (JDA-DAT) and other local governments [15]. Before data collection, dietitians of the prefectural government distributed the written instruction of how foods should be weighed and pictured and verbally explained the procedures to the qualified dietitians who were responsible for the records. Some of them were trained members of JDA-DAT and others were those working for local governments and usually engaged in the annual National Health and Nutrition Survey as a normal work. For secondary use in this study, we obtained all the dietary data available from the government.

### Dietary data collection

Dietary data were collected via measurement of food weights and food photographs. Digital scales were used to weigh foods. Information on scales used was not available; however, scales that can weigh 1–2 kg maximum (e.g., KJ114, Tanita co., Tokyo, Japan) are generally used in Japan [16]. For picture taking, the prefectural government staff instructed the dietitians to place the meal on an A4 sheet of paper and take a picture to ensure that the nutritional facts and names of the products and manufacturers were clearly visible. Weight data and photos were available for 98 meals; however, 12 meals that consisted of hot meal services or boxed meals were excluded from the nutrient calculation since the weight of the dishes and ingredients were not recorded and there was no product website where its nutritional information was posted. Thus, 86 meals were used in the analyses.

### Nutrient calculation

A nutrient calculation software (Excel add-in Eiyou plus (Kenpakusha)) was used to calculate the contents of energy, protein, vitamins B_1_, B_2_, and C, and salt (sodium content was calculated from the salt content). These were the nutrients whose target values were shown in the Revised RV. The Standard Tables of Food Composition in Japan (8th revision) was used in the software. The nutritional values were calculated using the Japanese national tables in cases when the weight of food or ingredients were recorded, or by referring to the general recipe in the software. To estimate the nutritional value of commercial foods, we consulted Nutrition Facts labels on the photographs and nutritional information on the company’s websites when the product name was identified from the photograph. If portion size was recorded in milliliters, it was converted to grams using the volume-to-weight conversion table [16].

### Meal classification

**Fig. 2:**
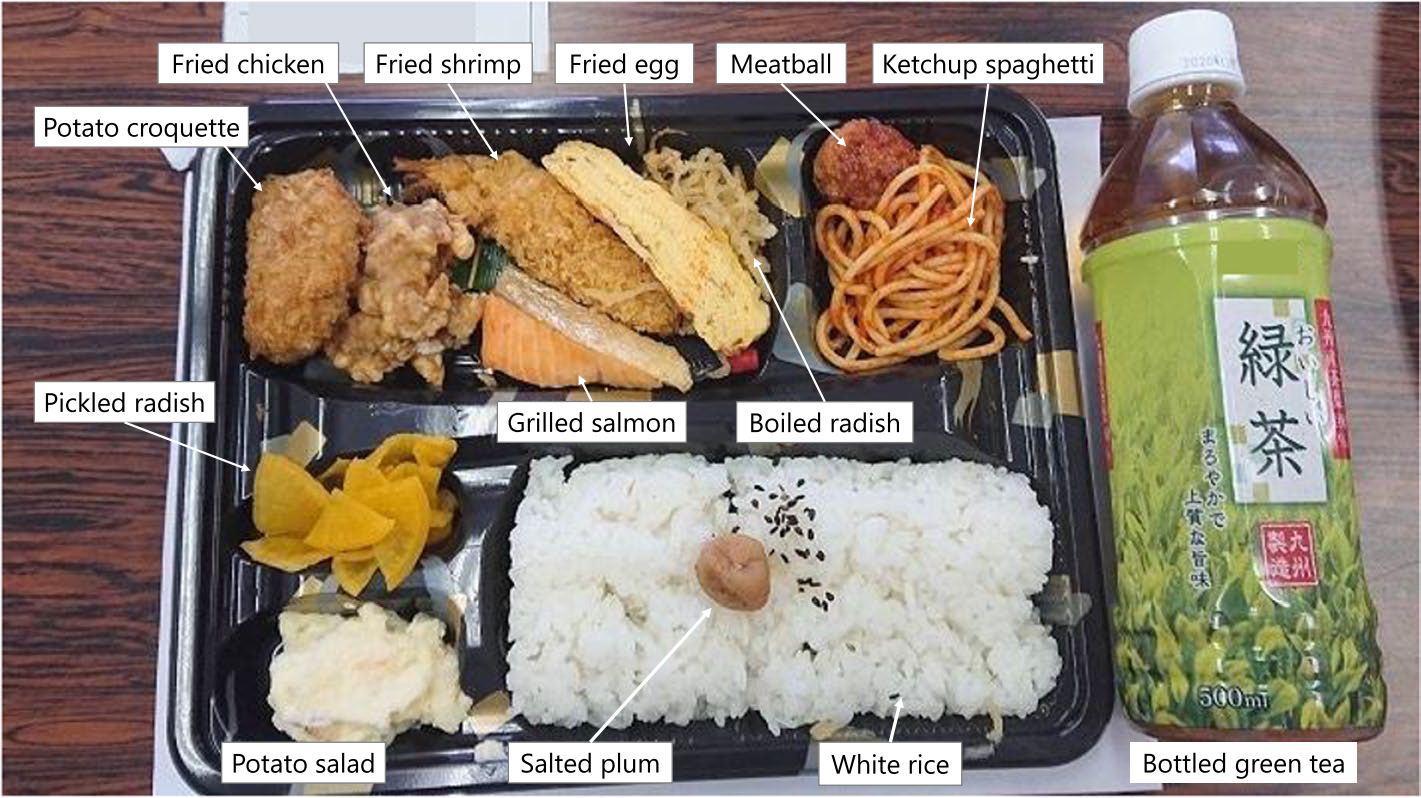
An example of “boxed meal with food aid” (provided on Day 17 as dinner). Boxed meal (left) and food aid (right; bottled green tea) were served together

**Fig. 3:**
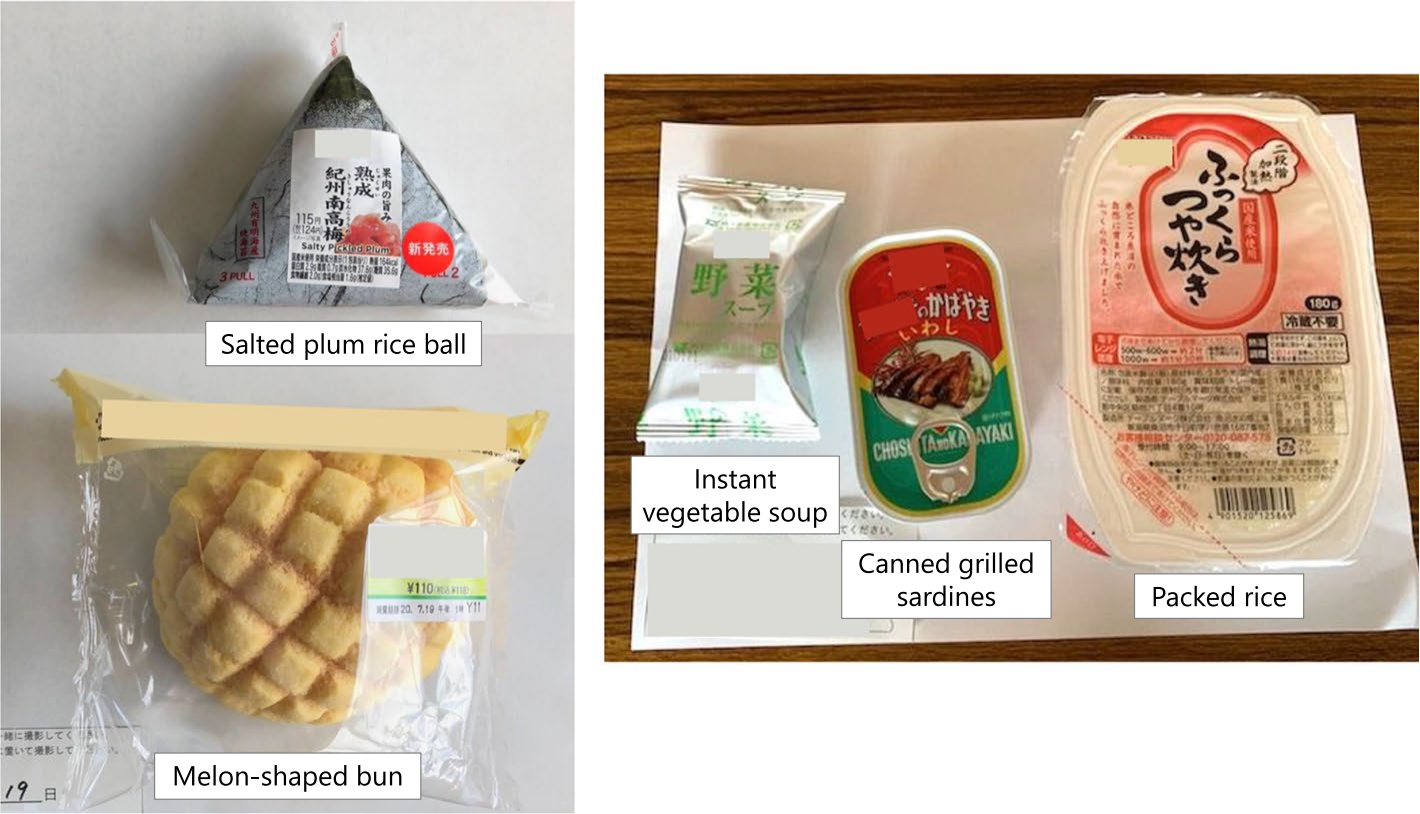
Two real examples of “food aid alone” meals. Left: Lunch on Day 16 (salted plum rice ball and melon-shaped bun). Right: Lunch on Day 19 (packed rice, canned grilled sardines, and instant vegetable soup)

**Fig. 4:**
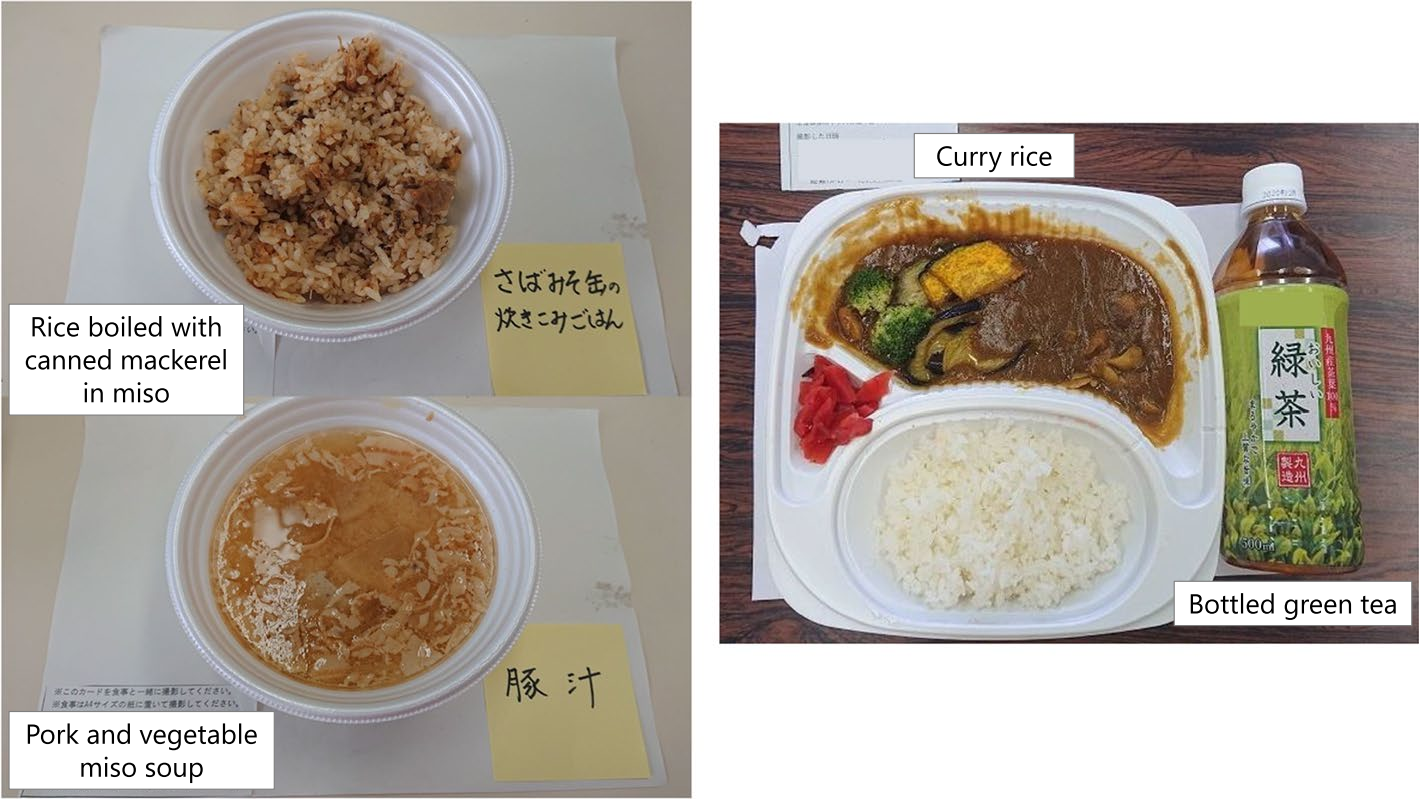
Two real examples of “hot meal service.”. Left: Dinner on Day 17 (rice boiled with canned mackerel in miso and pork and vegetable miso soup). Right: Dinner on Day 18 (curry rice and bottled green tea)

When recorded at shelters, each food was classified into four food types: boxed meal, food aid, hot meal service, and others. Boxed meals (Fig. 2) are ready-to-serve meals consisting of a staple and side dishes. Food aid (Fig. 3) is packaged commercially processed food. Hot meal services (Fig. 4) provide meals prepared by evacuees and/or aid organizations at shelters. No dishes were categorized as “others.” To analyze the nutritional differences by combination, meals were classified into six meal patterns: food aid alone, boxed meal alone, hot meal service alone, boxed meal with food aid, boxed meal with hot meal service, and hot meal service with food aid. The six meal patterns were organized into three groups according to the food category of staple food.

1. Meals consisting of food aid only (*n* = 32)

Seven kinds of foods were seen in the category of “food aid,” that is, rice ball, bread, rice, sandwich, protein, beverage, and others. Meals consisting of food aid only were classified into five subgroups according to combinations. In descending order of serving frequency, the five subcategories were bread only, rice ball + sandwich, rice ball + bread, rice + protein, and sandwich only.

2) Meals containing a boxed meal (boxed meal alone, boxed meal with food aid, and boxed meal with hot meal service) (*n* = 50)

Each dish included in boxed meals was classified into ten food groups as shown in “Examples of Food Composition Corresponding to the RV” by the National Institute of Health and Nutrition in Japan [9], according to its main ingredient. We subdivided vegetables and fruits into two groups: “pickled vegetables and fruits” and “vegetables (non-pickled).” The 11 subcategories were grains, fish and shellfish, meats, eggs, pulses, potatoes, pickled vegetables and fruits, vegetables (non-pickled), dairy products, fats and oils, and others.

3) Meals including food from hot meal service (hot meal service alone and hot meal service with food aid) (*n* = 4)

The four subcategories were mixed rice, white rice, soup, and others.

### Creation of menus

The target nutritional value of each menu was set at ≥ 1/3 of the Revised RV for energy, protein, and vitamins B_1_, B_2_, and C and < 1/3 of the Revised RV for salt.

1. Menu including food aid only

Based on frequently served food combinations, menus were developed by selecting nutritious foods from the same subcategories as the reference combinations. In cases when the nutrient content did not meet the target value, another food aid was added to supplement the deficient nutrient.

2) Menu including a boxed meal

The number of foods that comprised boxed meals was counted by subcategory. The menu including a boxed meal was created by combining the most frequently served or nutritious foods from each subcategory. When the nutritional value of boxed meal alone menu did not meet the target benchmark, addition of food aid or hot meal service was considered.

3) Menu including food from hot meal service

Foods with high nutrient or low salt content were selected from hot meal service. Food aids were added if hot meal service alone did not reach 1/3 of the Revised RV.

### Reproduction of foods served at shelters

We purchased the same or similar commercial foods recorded in the shelters. For boxed meals and foods from hot meal service, ingredients and seasonings were used in the same amounts as weighed at shelters. Since their cooking methods were unknown, we prepared the foods referring their photos.

### Cost calculation

The prices of food items used in the menus were surveyed at a major supermarket's online store in October 2022. The cost simulation for each menu was calculated by summing the price of each food item per weight.

### Statistical analysis

The Shapiro–Wilk test was performed to check distribution of each data. When intergroup difference was confirmed by the Kruskal–Wallis test, the Mann–Whitney U test was performed as post hoc analyses. Multiple comparisons were conducted with Bonferroni correction for type I errors. Significant level was set at 5%. All statistical analyses were performed using the IBM SPSS Statistics version 28.0 (IBM Corporation).

### Ethical considerations

This dietary survey was conducted by the Kumamoto prefectural government as a part of its backup operations for shelter management by municipalities in its jurisdiction. To use the data in this study, we obtained written permission for secondary use from the prefectural government. The names of the shelters and municipalities were anonymized in accordance with the agreement. It also allowed us to present food photos in our paper as long as the name of the manufacturer was not disclosed. As this is a secondary use of data already collected by the government, the Ethical Review Committee of the Humanities and Social Sciences Studies of the Ochanomizu University decided that it was not subject to research ethics review.

## Results

### Characteristics of shelters

The number of evacuees per shelter ranged 15–300 people. Although age groups of the evacuees were not recorded in the sheet, infants were identified by the check column for vulnerable people. In total, records for 32 days and 98 meals were available: the number of meals served per day was twice for 2 days and three times for 30 days.

### Meals served in shelters

None of the meals served in the shelters satisfied the target (1/3 of the Revised RV).

Table 1 shows the number of meals that met the target for each nutrient through a combination of foods (meals including food aid alone meals) or meal pattern (meals containing boxed meals, and meals including hot meal service). Additionally, the number of appearances for each food is tabulated by subcategory in Table 2.

**Table 1:** The number of meals that met 1/3 of the Revised RV for each nutrient

Meal group (n)^a^	Component of the meal (n)^a^	The number of meals that met 1/3 of the Revised RV					
		Energy [≥ 667 kcal]	Protein [≥ 18.3 g]	Vitamin B_1_ [≥ 0.3 mg]	Vitamin B_2_ [≥ 0.33 mg]	Vitamin C [≥ 27 g]	Salt [< 2.7 g of salt (1063 mg of sodium)]
**Meals including food aid alone (32)**	Bread alone (6)	1	0	0	1	1	6
	Rice ball + sandwich (5)	0	0	2	0	1	3
	Rice ball + bread (5)	1	0	0	0	0	4
	Rice + protein (3)	0	3	1	0	0	0
	Sandwich only (3)	0	0	0	0	0	3
	Others (10)	1	1	1	0	0	4
**Meals containing boxed meal (50)**	Boxed meal alone (32)	14	25	6	6	1	16
	Boxed meal with food aid (14)	5	14	4	6	10	5
	Boxed meal with hot meal service (4)	4	4	3	3	2	0
**Meals including hot meal service (4)**	Hot meal service alone (3)	1	1	1	1	0	0
	Hot meal service with food aid (1)	0	0	0	1	1	1

^a^The number of meals that appeared

**Table 2:** The number of times food items were served in each subcategory by meal group

Meal group [n]^†^	Food type [n]^†^	Food group [n]^†^ (n)^‡^	Names of food (n)^‡^
**Meals consisting of food aid alone ** [32]	Food aid [32]	Rice ball [13] (17)	Simmered kelp (8), Salted plum (5), Salmon (3), Finely chopped katsuobushi with soy sauce (1)
		Bread [12] (12)	Melon-shaped bun (4), Strawberry jam and margarine bun (4), Curry doughnut (1), Danish pastry (1), Strawberry jam bread (1), Canned bread (1)
		Rice [9] (10)	Packed rice (5), Preprocessed rice (5)
		Sandwich [8] (8)	Ham and cheese (2), Egg and tuna (2), Egg, ham and tuna (1), Cutlet (1), Ham (1), Egg (1)
		Protein [3] (3)	Canned sardine (2), Packaged tofu (1)
		Beverage [5] (5)	Vegetable juice (2), Bottled green tea (1), Lactic acid drink (1), Sports drink (1)
		Others [9] (9)	Instant soup (6), Cup-type instant noodle (2), Retort-pouch curry (1)
**Meals containing a boxed meal [50]**	Boxed meal [50]	Grains [50] (82)	White rice (36), Spaghetti (17), Rice ball (8), Rice with laver (4), etc
		Fish and shellfish [33] (39)	Grilled mackerel (11), Grilled salmon (8), Fried shrimp (7), Fried fish (6) etc
		Meats [40] (65)	Fried chicken (18), Steamed meat dumpling (8), Meat ball (8), Wiener (7) etc
		Eggs [38] (40)	Fried egg (27), Fried egg with vegetables (6) etc
		Pulses [16] (17)	Boiled beans (10), Tofu hamburger steak (3) etc
		Potatoes [29] (35)	Potato croquette (17), Potato salad (12) etc
		Vegetables (non-pickled) [37] (80)	Cabbage (14), Simmered kiriboshi-daikon (6), Braised burdock root (4), Simmered kelp (3) etc
		Pickled vegetables and fruits [33] (45)	Pickled radish (22), Salted plum (17) etc
		Others [14] (14)	Simmered hijiki (seaweed) (8) etc
	Food aid [14]	Beverage [11] (13)	Lactic acid drink (5), Vegetable juice (4), Bottled green tea (3), Milk with long shelf-life (1)
		Others [3] (3)	Orange jelly (2), Cup-type instant noodle (1)
	Hot meal service [4]	Soup [4] (4)	Chinese style soup with tofu and onions (1), Consommé soup with cabbage and potatoes (1), Consommé soup with cabbage and onions (1), Chicken and vegetable miso soup (1)
**Meals including hot meal service ** [4]	Hot meal service [4]	Mixed rice [3] (3)	Bamboo shoots rice (1), Rice boiled with canned mackerel in miso (1), Hijiki (seaweed) rice (1)
		White rice [1] (1)	Curry rice (1)
		Soup [2] (2)	Pork and vegetable miso soup (1), Chicken meatball soup (1)
		Others [2] (6)	Carrot stir-fry (1), Cabbage stir-fry (1), Meatball (1), Pickled ginger (1), potato salad (1), and Kikurage (wood ear mushroom) topped with egg (1)
	Food aid [1]	Beverage [1] (1)	Bottled green tea (1)

^†^The number of meals that appeared

^‡^The total number of each food item provided

Example: Rice ball [13] ^†^(17)^‡^

^†^Meal that included rice ball was observed 13 times

^‡^A total of 17 rice balls was provided at 13 meal occasions

#### Meals consisting of food aid only

**Fig. 5:**
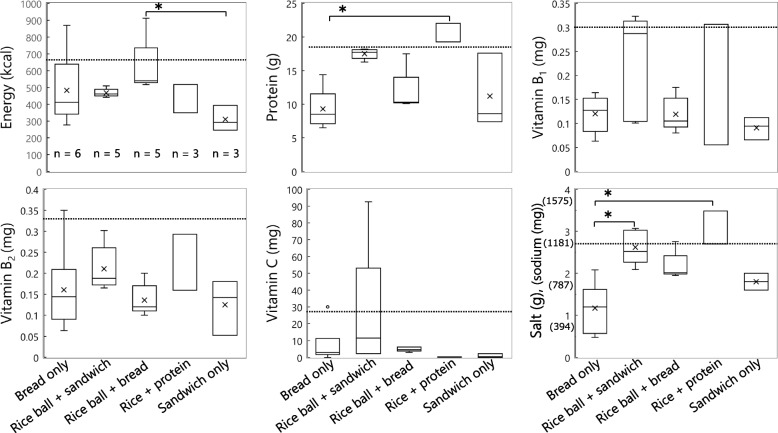
Nutritional profiles of five most frequently served combinations in food aid only meals. Bread only (*n* = 6), rice ball + sandwich (*n* = 5), rice ball + bread (*n* = 5), rice + protein (*n* = 3), and sandwich only (*n* = 3). × : Mean value for normal distribution. ○: outlier (values farther than 1.5 times the interquartile range). Dotted line: 1/3 of the Revised RV. *: Mann–Whitney U test, *p* < 0.05

Table 1 shows that 26 of 32 meals met the criterion for salt (< 2.7 g of salt (1063 mg of sodium)); however, only one meal exceeded 1/3 of the Revised RV for vitamin B_2_. Frequently served meals were bread only (*n* = 6), rice ball + sandwich (*n* = 5), rice ball + bread (*n* = 5), rice + protein (*n* = 3), and sandwich only (*n* = 3). As shown in Fig. 5, bread-only meals were significantly low in protein and salt content. Only a meal with two pieces of bread exceeded 1/3 of the Revised RV in energy. A meal (melon-shaped bun + bottled green tea) met the target for vitamins B_2_ and C owing to the inclusion of bottled green tea. In the rice ball + sandwich combination, two meals including the ham and cheese sandwich had > 1/3 of the Revised RV for vitamin B_1_; however, these two meals contained excessive amounts of salt. Vegetable juice contributed to meeting the target for vitamin C in the rice ball + sandwich combination. In the rice ball + bread combination, only the rice ball and two pieces of bread provided more energy (912 kcal) than the reference value. All meals containing the combination of rice + protein provided > 1/3 of the Revised RV of protein; however, they exceeded the reference value in terms of the salt content. A meal containing tofu also exceeded 1/3 of the Revised RV for vitamin B_1_. Sandwich only did not meet the criteria for nutrients other than salt.

#### Meals containing a boxed meal

**Fig. 6:**
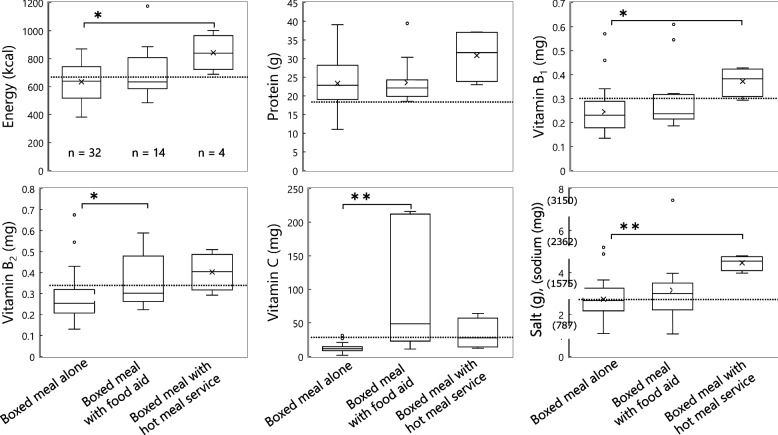
Nutritional profiles of 50 meals containing boxed meal by meal pattern; boxed meal only (*n* = 32), boxed meal with food aid (*n* = 14), and boxed meal with hot meal service (*n* = 4). × : Mean value for normal distribution, ○: outlier (values farther than 1.5 times the interquartile range), the dotted line in each graph: the amount of 1/3 of the Revised RV. Mann–Whitney U test for energy, vitamins B_1_, B_2_, and C, and salt, *: *p* < 0.05, **: *p* < 0.01

In the “boxed meal with food aid” meals, one or two varieties of beverages, instant noodles, or orange jelly were provided as food aid (Table 2). In “boxed meal with hot meal service,” a boxed meal and a cup of soup were served together (Table 2). Evacuees could not choose food aid or soup. Vitamins B_2_ and C were significantly higher in “boxed meal with food aid” than in “boxed meal alone” (Fig. 6). Among the food aids, milk and bottled green tea were rich in vitamin B_2_, whereas vegetable juice, bottled green tea, and orange jelly were high vitamin C content. When a boxed meal and a cup-type instant noodle were served together, energy (> 1,000 kcal per meal) and salt content (7.4 g) far exceeded one-third of the Revised RV. “Boxed meal with hot meal service” provided significantly more energy, vitamin B_1_, and salt than “boxed meal alone,” in particular, energy and salt content was considerably higher than 1/3 of the Revised RV (Fig. 6).

#### Meals including food from hot meal service

As shown in Table 1, “hot meal service alone” did not meet the criteria for vitamin C and salt, and “hot meal service with food aid” (curry rice with bottled green tea) were insufficient in terms of energy, protein, and vitamin B_1_. Among staples served as part of the hot meal service, all mixed rice meals contained higher nutrients than white rice; however, their salt contents were also higher. Both soups containing vegetables and meat supplied approximately 1/3 of the target values for protein and vitamins B_1_, B_2_, and C per serving.

### Creation of menus

Five menus were devised using only foods served in the shelters. In most cases, the planned menus contained more energy, protein, and vitamins than the actual meals, and less salt. Among them, vitamin C was higher than 1/3 of the Revised RV in the menus.

#### Menu A (Food Aid Only) = Curry Doughnut + Milk with long shelf-life + Orange Jelly (Fig. 7)

**Fig. 7:**
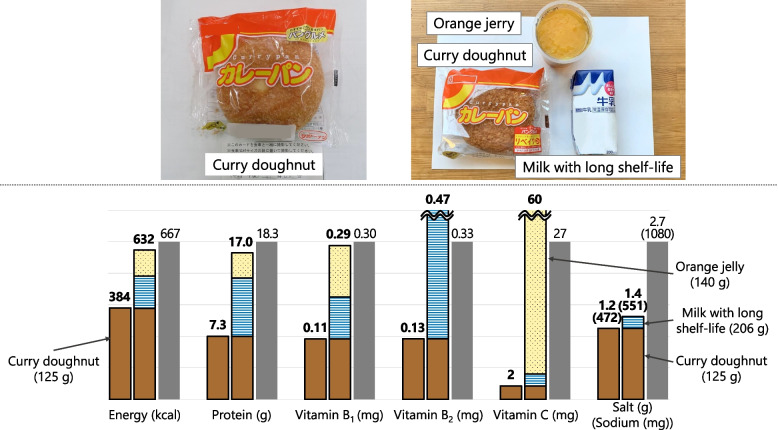
Comparison of nutrient contents between food served at shelters (real case) and planned Menu A. Colored bars show nutrient contents in real case (left) and Menu A (right). Gray bar shows 1/3 of the Revised RV. Real case = curry doughnut (brown). Menu A = curry doughnut (brown) + milk with long shelf-life (blue) + orange jerry (yellow)

The “bread only” meal was considered the foundation as it was most frequently served among the food aid alone meals. Furthermore, curry doughnut alone did not reach 1/3 of the Revised RV, although it contained relatively high amounts of energy and protein among bread. We added milk and orange jelly to increase the supply of protein, and vitamins B_2_ and C.

#### Menu B (Food Aid Only) = Salmon Rice Ball + Ham and Cheese Sandwich + Vegetable Juice (Fig. 8)

**Fig. 8:**
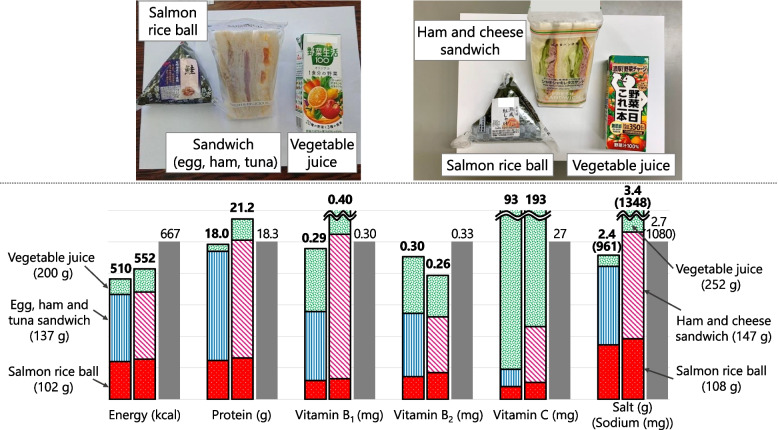
Comparison of nutrient contents between food served at shelters (real case) and planned Menu B. Colored bars show nutrient contents in real case (left) and Menu B (right). Gray bar shows 1/3 of the Revised RV. Real case = salmon rice ball (red) + egg, ham and tuna sandwich (blue stripes) + vegetable juice (green). Menu B = salmon rice ball (red) + ham and cheese sandwich (pink stripes) + vegetable juice (green)

This menu was created based on “rice ball + sandwich” because it often was the second-most served meal after “bread only,” and had a higher median value of nutrients except energy than the “rice ball + bread” combination (Fig. 5). We referred to the “salmon rice ball + egg, ham, and tuna sandwich + vegetable juice” meal that contained most energy, vitamins B_2_ and C. Salmon rice ball had the highest content of all nutrients among the rice balls and vegetable juice was high vitamin C content; we adopted this combination. Regarding the sandwich, ham and cheese provided more energy, protein, and vitamins B_1_ and C than egg, ham, and tuna (the bar graph in Fig. 8); thus, ham and cheese sandwich was used in the menu.

#### Menu C (Boxed Meal with Food Aid) = Boxed Meal + Vegetable Juice (Fig. 9)

**Fig. 9:**
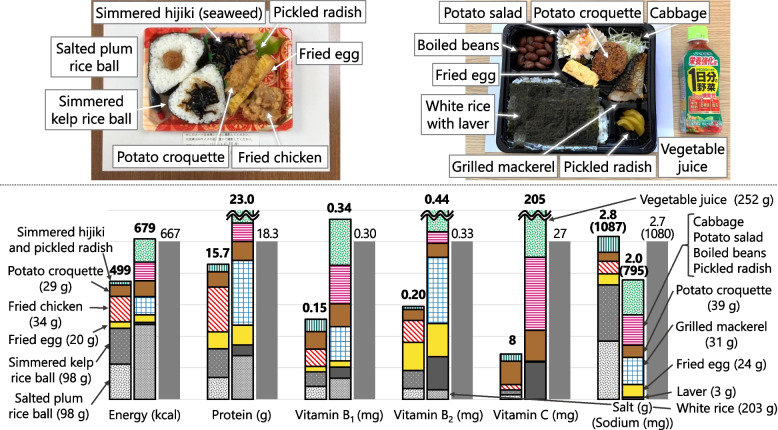
Comparison of nutrient contents between food served at shelters (real case) and planned Menu C. Colored bars show nutrient contents in real case (left) and Menu C (right). Gray bar shows 1/3 of the Revised RV. Real case = salted plum rice ball (gray) + simmered kelp rice ball (gray) + fried egg (yellow) + fried chicken (orange stripes) + potato croquette (brown) + simmered hijiki and pickled radish (green). Menu C = white rice (gray) + laver (dark gray) + fried egg (yellow) + grilled mackerel (blue) + potato croquette (brown) + cabbage, potato salad, boiled beans, and pickled radish (pink stripes) + vegetable juice (green)

The typical contents of boxed meals were as follows: one kind of grains for staple food (44% of 50 boxed meals); more than two kinds of eggs, meats, or fish and shellfish for main dishes (86%); 2–5 side dishes of pulses, potatoes, vegetables (non-pickled), or pickled vegetables and fruits (76%). No dairy products or fats and oils were added in the boxed meals (Table 2). White rice was used in the planned menu because it was the most served staple food, as shown in Table 2. Furthermore, we added laver to increase vitamin B_2_ and C contents. Fried egg was chosen because it was served most frequently in the eggs, meats, and fish food groups (Table 2). Comparing grilled mackerel with fried chicken, which were the most frequently served main dishes after fried eggs, grilled mackerel appeared to have more protein, vitamins B_1_, and B_2_ than fried chicken for the same amount; we included grilled mackerel in the menu considering this point. Potato croquette, cabbage, potato salad, boiled beans, and pickled radish were used because they were frequently served as side dishes. Salted plum was excluded because its nutrient contents were few except for salt. We then added vegetable juice to supplement energy and vitamins.

#### Menu D (Hot Meal Service with Food Aid) = Chicken Meatball Soup + Packaged Tofu + Soy Sauce + Preprocessed White Rice + Bottled Green Tea (Fig. 10)

**Fig. 10:**
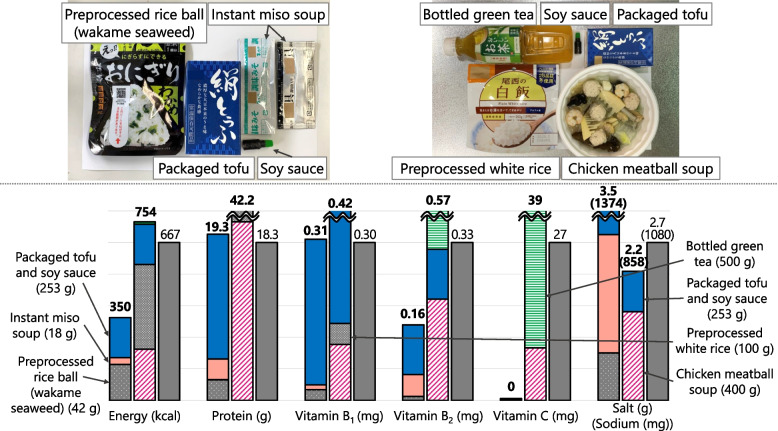
Comparison of nutrient contents between food served at shelters (real case) and planned Menu D. Colored bars show nutrient contents in real case (left) and Menu D (right). Gray bar shows 1/3 of the Revised RV. Real case = preprocessed rice ball (wakame seaweed) (dotted gray) + instant miso soup (pink) + packaged tofu and soy sauce (navy). Menu D = chicken meatball soup (pink stripes) + preprocessed white rice (gray) + packaged tofu and soy sauce (navy) + bottled green tea (green stripes)

The chicken meatball soup had the highest energy, protein, and vitamin B_2_ among the soups served in the hot meal service. Since soup alone did not reach 1/3 of the Revised RV for energy and vitamin B_1_, we added preprocessed white rice and packaged tofu collected from food aids. Moreover, vitamins B_2_ and C were increased by adding bottled green tea.

#### Menu E (Hot Meal Service with Food Aid) = Bamboo Shoots Rice + Chicken and Vegetable Miso Soup + Bottled Green Tea (Fig. 11)

**Fig. 11:**
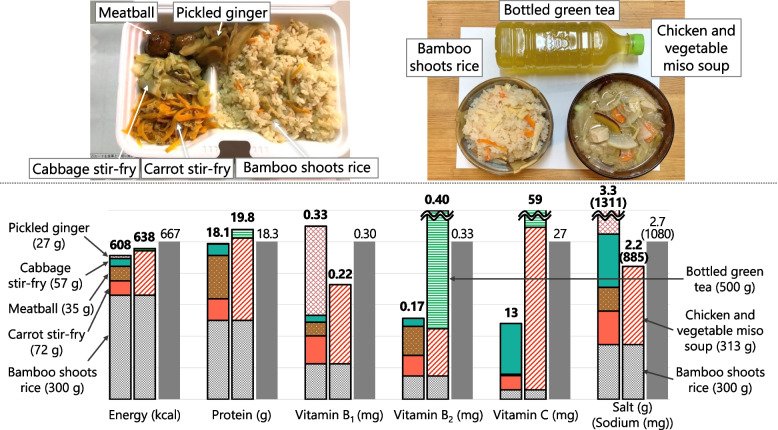
Comparison of nutrient contents between food served at shelters (real case) and planned Menu E. Colored bars show nutrient contents in real case (left) and Menu A (right). Gray bar shows 1/3 of the Revised RV. Real case = bamboo shoots rice (gray) + carrot stir-fry (orange) + meatball (brown) + cabbage stir-fry (green) + picked ginger (red). Menu E = bamboo shoots rice (gray) + chicken and vegetable miso soup (orange stripes) + bottled green tea (green stripes)

Bamboo shoots rice was selected because it had the lowest salt and highest vitamin B_1_ content among mixed rice of hot meal service. The amount of vitamin C in the chicken and vegetable miso soup was > 1/3 of the Revised RV per serving and was the richest in energy, protein, and vitamin B_2_ next to the chicken meatball soup; thus, we combined it with bamboo shoots rice. Bottled green tea was added to supplement vitamin B_2_.

#### Example of a combination of three menus for daily meal planning

**Fig. 12:**
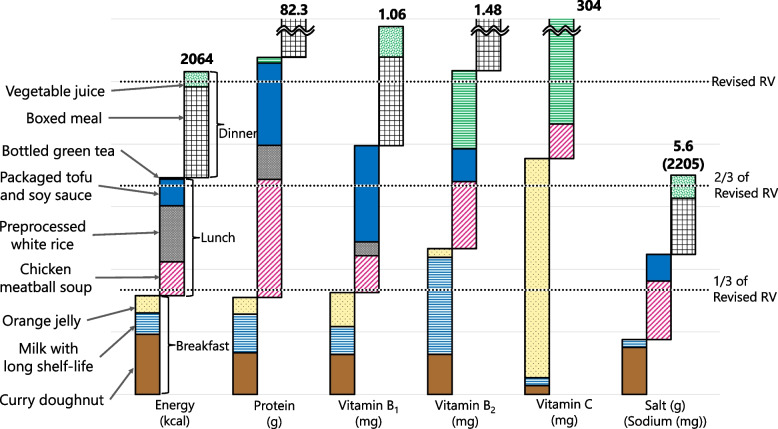
An example of a daily menu using the Menus A (curry doughnut, milk with long shelf-life, and orange jelly), C (boxed meal and vegetable juice), and D (chicken meatball soup, preprocessed white rice, packaged tofu and soy sauce, bottled green tea). Three dotted lines represent 1/3, 2/3, and 3/3 of Revised RV from the bottom

Figure 12 presents an example of a daily menu using the Menus A, C, and D. In this example, all nutrients satisfied the daily requirements according to the Revised RV.

### Cost estimation

All menus were estimated to cost more than the actual meals (Table 3).

**Table 3:** Comparison of costs between real cases and planned menus

**Menu**		**Contents**	**Cost (¥)**
**A**	Real case	curry doughnut	110
	Planned menu	curry doughnut, milk with long shelf-life, and orange jelly	315
**B**	Real case	salmon rice ball, egg, ham and tuna sandwich, and vegetable juice	568
	Planned menu	salmon rice ball, ham and cheese sandwich, and vegetable juice	570
**C**	Real case	salted plum rice ball, simmered kelp rice ball, fried egg, fried chicken, potato croquette, simmered hijiki, and pickled radish	242
	Planned menu	white rice, laver, fried egg, grilled mackerel, potato croquette, cabbage, potato salad, boiled beans, pickled radish, and vegetable juice	410
**D**	Real case	preprocessed rice ball (wakame seaweed), instant miso soup, packaged tofu, and soy sauce	379
	Planned menu	chicken meatball soup, preprocessed white rice, packaged tofu, soy sauce, and bottled green tea	601
**E**	Real case	bamboo shoots rice, carrot stir-fry, meatball, cabbage stir-fry, and picked ginger	263
	Planned menu	bamboo shoots rice, chicken and vegetable miso soup, and bottled green tea	318

## Discussion

In the evacuation shelters, 16–20 days after the flood, three types of foods were provided: food aid, boxed meals, and hot meal service. Since none of 86 meals served met 1/3 of the Revised RV, we developed five different menus that supplied energy and nutrients near or above the Revised RV by choosing nutritious foods within the same category or by adding a vitamin-rich beverage or jelly.

### Revised RV as a target value

Since the Revised RV was used as the target value for menu planning, we were unable to consider lipids, minerals, and other vitamins shown in the Dietary Reference Intakes for Japanese. In a previous study, local government dietitians with experience of nutrition assistance in disaster-affected areas suggested that the Revised RV should be minimal as it was difficult to meet the original RV in the past disasters [10]. Therefore, the Revised RV that limited the number of nutrients might be realistic.

### Characteristics by food categories

#### Food Aid

In the present study, carbohydrates such as rice balls, bread, and rice made up the majority of food aids, whereas protein sources and beverages were provided less frequently. This was also observed in evacuation shelters in the past; 1 month after the Great East Japan Earthquake, food aid predominantly comprised of carbohydrates such as rice balls and bread [17]. Some shelters (16%) were oversupplied with carbohydrates, especially sweet bun [3]. As shown in Figs. 7 and 8, the meals consisting of bread, rice balls, and sandwiches were particularly low in vitamins and their supplies were far below the reference values (1/3 of the Revised RV). Our results have demonstrated that providing milk, orange jelly, and vegetable juice can ensure that the amount of vitamins reach the reference levels even when bread and rice balls are provided as a staple. In this study, the nutritional value of bottled green tea was estimated on the basis of the value of green tea without added vitamins, as listed in the Standard Tables of Food Composition in Japan. However, vitamin C is typically added to prevent degradation of catechins, and commercial bottled green tea in Japan is reported to contain 425–1852 µg/mL of vitamin C (213–926 mg/500 mL (per bottle)) [18]. Therefore, the bottled green tea served in the shelters may contain a higher amount of vitamin C than the value estimated in the present study. Bottled green tea in Japan is generally unsweetened. In a shelter in the past, many people said that they like drinking tea with their meals [19]; thus, the provision of bottled green tea with meals meets the needs of evacuees in terms of not only nutrition but also preference.

#### Boxed meals

Approximately 80% of all boxed meals supplied protein more than 1/3 of the Revised RV, whereas few boxed meals supplied vitamins > 1/3 of the Revised RV. These results are generally consistent with previous studies demonstrating that the supply of energy and protein became significantly higher when a boxed meal was provided, whereas vitamins B_1_ and C contents were significantly lower [20]. More than half of the boxed meals contained salt above the target value. A previous study on 20 commercial boxed meals reported a mean value of 3.8 g of salt contained per box [21]. Therefore, excessive salt intake from boxed meals should be taken into consideration. Furthermore, as observed in disaster areas in the past [22], concern regarding excessive energy and salt intake is evident when a boxed meal and a soup of hot meal service are served together. Owing to the fact that sodium intake was significantly associated with disaster hypertension [23], providing vitamin-rich food aid such as vegetable juice, bottled green tea, and orange jelly would be recommended rather than a hot meal service. This is also applicable in cases when the amount of vitamin is inadequate with only a boxed meal. In the planned menu C, mackerel provided more protein and vitamins B_1_ and B_2_. Seafood is the second most prominent food after green vegetables that evacuees wanted to eat more [24], and a significant association was observed between its consumption frequency and becoming easily irritated [25]. Therefore, including fish in a boxed meal may not only increase its nutritional value but also increase meal satisfaction among evacuees and reduce complaints.

#### Hot meal service

In the past disasters, it has been pointed out that the amount of vegetables provided in shelters was small [3, 26], and the disaster victims wanted to eat more [26–28]. Morishita and Kubo [28] reported that there were many requests for hot meals in shelters, and vegetable soup provided by hot meal service, like our chicken and vegetable miso soup, is expected to improve their meal satisfaction. According to a nationwide survey in Japan, although quantities were not shown, > 80% of municipalities stockpiled staple foods with long-shelf life at room temperature, such as preprocessed rice and canned bread, whereas only < 20% of them stockpiled side dishes (canned or retort-pouch foods with long-shelf life at room temperature) [29]. Staple (preprocessed rice and dried bread) alone cannot meet the Revised RV, and vitamins in particular are far below the recommended target [30]. Owing to the fact that the amount of vitamins increased significantly through hot meal service [20], even if only staple foods are stockpiled, nutrient supplies may be closer to the recommendation by providing hot meal service, as in Menu D.

### Feasibility of planned menus

#### Menu A

As shown in Table 4, bread was served at an early point after disasters. Milk with a long shelf-life was delivered by the central government about two weeks after the Kumamoto Earthquake [31]. Our records confirmed its delivery 18 days after the disaster as well. Although we could not confirm if orange jelly was included in the items of the push-type support from the government, it was provided in two shelters 20 days after the disaster in this survey. Judging from these facts, we suppose that a meal consisting of bread and milk can be provided in about 2 weeks after the disaster, and a meal with an orange jelly can be provided in about 3 weeks.

**Table 4:** The first day of serving foods used in planned menus in past disasters

Menu	Food	The earliest day of serving	The name of the disaster (occurrence year) [reference number]
**A**	Bread (Data of curry doughnut is not available)	Day 3 after the foreshock	Kumamoto Earthquake (2016) [31]
		Day 2	Hokkaido Eastern Iburi Earthquake (2018) [32]
	Milk with long-shelf life	Approximately 2 weeks later	Kumamoto Earthquake (2016) [31]
	Orange jelly	Day 20	Heavy Rain Event in July 2020 (2020) (present study)
**B**	Rice ball	Day 2 after the foreshock	Kumamoto Earthquake (2016) [33]
	Sandwich	Day 17	Heavy Rain Event in July 2020 (2020) (present study)
**B, C**	Vegetable juice	Day 2	Hokkaido Eastern Iburi Earthquake (2018) [32]
		1 week later	Heavy Rain Event in July 2020 (2020) [34]
**C**	Boxed meal	Day 12	Great East Japan Earthquake (2011) [35]
		Approximately 1.5 months later	Kumamoto Earthquake (2016) [33]
**D**	Preprocessed rice	Day 2	Kumamoto Earthquake (2016) [36]
	Packaged Tofu	Day 20	Heavy Rain Event in July 2020 (2020) (present study)
**D, E**	Hot meal service	Day 1	Great East Japan Earthquake (2011) [37]
		Day 4	Kumamoto Earthquake (2016) [36]
	Bottled green tea	Day 5	Hokkaido Eastern Iburi Earthquake (2018) [32]
		Day 4	Heavy Rain Event in July 2020 (2020) [34]

#### Menu B

Rice ball and vegetable juice were included in the push-type support by the government during the past disasters; they were supplied two days after disasters [32, 33] (Table 4). In this study, sandwiches were served at several shelters 17 days after the disaster. Although we were unable to confirm when sandwiches were served in other disasters, it is estimated that a meal consisting of a rice ball, sandwiches, and vegetable juice can be served within 1–3 weeks after a disaster.

#### Menu C

The start of serving boxed meals ranged from 12 days to 1.5 months depending on the disasters [33, 35]. In the case of recent heavy rain disaster, boxed meals were served on the first day of the survey, i.e., 16 days after the disaster occurred. Croquettes, potato salad, mackerel, and fried eggs used in Menu C were frequently used in convenience stores’ boxed meals [21]. Because Menu C used the standard ingredients of boxed meals sold in normal times, when food distribution is restored, preparing side dishes according to our menu might be relatively easy. Considering that vegetable juice was actively sent to disaster areas, a set of a boxed meal and vegetable juice could be served approximately 2 weeks after a disaster.

#### Menus D and E

Hot meal service started on the day of the disaster; however, simple meals such as rice balls were served in the early post-disaster period. Hot meal service with fresh foods, such as vegetables and meat, was provided at multiple shelters 16 days after the disaster in our survey. Because storage is an issue when using perishable foods, availability of refrigerators and freezers expanded the variety of foods provided [37]. These electrical goods were delivered to the disaster areas about one week after the Heavy Rain Event in July 2020 by the government [34]. Bottled green tea was sent to the affected areas four days after the flood disaster where the data of this study was collected [34]. Preprocessed rice is suitable for stockpiling and was available the day after the past earthquake [36]. Packaged tofu that needs not be refrigerated did not appear as an item for push-type support. However, it is recommended by the Ministry of Agriculture, Forestry and Fisheries for household stockpiling as it can be stored for a long time at room temperature [38]. If people know the usefulness of this food in times of disaster, it will possibly be stockpiled and provided as food aid in future disasters. For menus E and D, bottled green tea and preprocessed rice are likely to be served relatively early, and hot meal service using fresh food can be provided about two weeks after a disaster.

### Costs of planned menus

The cost of all menus was estimated to be higher than that of actual meals. At the time of a disaster in Japan, food expenses are subsidized based on the Disaster Relief Act for those who evacuate to shelters or are unable to cook owing to a disaster. The general standard is now ¥1,180 per person per day [39]; however, special addition can be applied when nutritious meals could not be provided by that amount of money. In the Great East Japan Earthquake, while the general standard was ¥1,010 at that time, the total amount was raised up to ¥1,500 per person per day [37]. For the menu in this study, the most expensive daily meal plan (Menus B, C, and D) cost ¥1,581, and the cheapest combination (Menus A, C, and E) was ¥1,043. Many combinations exceeded the current general standard of ¥1,180; however, if special addition is allowed as in the past disasters, our menus can be provided without municipality’s expense.

### Users of model menus

In past disasters, menu preparation and advice were one of the major support activities of dietitians. After the Great East Japan Earthquake, dietitians proposed menus for the victims and the Self-Defense Forces who provided hot meal service [12, 40]. In addition, at the time of the Kumamoto Earthquake, menu planning support accounted for approximately 70% of JDA-DAT's support activities at shelters [33]. Our menus that satisfy the Revised RV may reduce the burden on dietitians for considering nutritionally balanced menus in disasters. Furthermore, owing to the fact that our menus consisted of only foods available at past shelters, they have a relatively high possibility to be realized in future disasters.

### Limitations

This study has several limitations. First, the dietary data was collected for a limited period of 16–20 days after a single heavy rain disaster. Although similar foods were provided and similar nutritional problems were repeated in the past disasters, commencing time of provision of boxed meals and hot meal service varied depending on the type and severity of the event, status of lifelines, and affected areas. There is a possibility that provision of our menus might be delayed in other harsh situations. Second, data collection was not research-based, and the scientific sampling technique was not used because the dietary survey was conducted as a part of public services to improve the conditions of shelters run by municipalities. Third, although direct measurement was employed, weights of some foods, such as miso used in miso soup, were difficult to weigh, resulting in some errors. Finally, individual needs regarding food allergies or dietary restrictions were not considered when developing menus.

## Conclusions

Although none of the meals actually served at the shelters met the recommended levels (1/3 of the Revised RV), we devised five menus that supplied energy and nutrients near or above the reference values by using frequently served or nutritious foods in the past shelters. According to Japan’s past disaster experiences, all menus were estimated to be provided within 1–3 weeks after disasters. This study demonstrated that menus with nutritional profiles similar to or higher than the Revised RV can be developed by changing the combination of foods available in the shelters.

## Supplementary Information

**Additional file 1.**

## Data Availability

The data are not publicly available due to the contract with the Kumamoto prefectural government. However, the data are available from the corresponding author on reasonable request.

## Abbreviations

RV: Nutritional Reference Values for dietary planning at emergency shelters
Revised RV: Revised Nutritional Reference Values for Feeding at Evacuation Shelters
MHLW: Ministry of Health, Labour and Welfare
JDA-DAT: The Japan Dietetic Association-Disaster Assistance Team
