# Supplementary material for: Meal plans for meeting the reference values using food items available in shelters
Source: BMC Nutr. 2023 Jun 24;9:73. doi: 10.1186/s40795-023-00726-9 (PMC10290345; doi:10.1186/s40795-023-00726-9)
Supplement: Supplementary file 1 — Additional file 1. [file 40795_2023_726_MOESM1_ESM.docx]

| Survey date | | MM/DD/YYYY (Day of the week) | | | | | Person who filled out the form | Your organization | | □Public Health Center  □Municipality  □Other Municipality | |
| --- | --- | --- | --- | --- | --- | --- | --- | --- | --- | --- | --- |
|  |  |  |  |  |  |  |  |  |  | □Association of Dietitians  □Other： | |
|  |  |  |  |  |  |  |  | Name | |  | |
| Name of Evacuation Shelter | |  | | | | | Category of evacuation shelter | □ Designated　□ Other： | | | |
| Number of evacuees | | Evacuees: Total ( ) persons  【 □ < 50 persons □ 51 to 100 persons □101 to 150 persons □151 to 500 persons □ > 501 persons】  Number of meals for evacuees (evacuees at home) who only come to get meals: ( ) meals | | | | | | | | | |
| Person in charge | | Name ： | | | | Your position | □Person in charge of shelter  □Person in charge of meal provision  □Others: | | | | |
| Number of meals served | | □ None　□ Once　□ Twice  □ Three times /day | | | | | Drinking water | □ None □ Insufficient (less than 1.5L per person per day) □ Sufficient | | | |
| Check the box for the persons in need of consideration at the shelter ☑  * Just check the box if it is difficult to grasp the number of people | | □Infants | | | persons | Check the ones that are lacking☑ | □Infant formula □Weaning food　□Diapers □Other： | | | | |
|  |  | □Food allergy | | | persons |  | □7 items removed from diet □Elimination of foods other than the 7 items (causative food: ) | | | | |
|  |  | □Hypertension | | | persons |  | □Low-sodium diet　□Antihypertensive drug　□Other： | | | | |
|  |  | □Diabetes | | | persons |  | □Energy regulated diet □Medication　□Insulin □Other： | | | | |
|  |  | □Kidney disease | | | persons |  | □Low protein diet　□Low protein diet　□Medicine　□Other： | | | | |
|  |  | □Person who has difficulty swallowing and eating | | | persons |  | □Thickening adjustment food　□Food for dysphagic person　□Other: | | | | |
|  |  | □Pregnant Breastfeeding woman | | | persons |  | | | | | |
|  |  | □Other:  □No one in need of care  Lifeline utilities available | | | |  |  |  |  |  |  |
| Lifeline utilities available | | □Electricity | | | | | | □Waterworks | | | |
|  |  | □Gas （Boiled water） | | | | | | □Sewerage | | | |
|  |  | □Access to people and goods by car | | | | | | □Swimming pool water | | | |
|  | |  | | | | | |  | | | |
| General meals served at evacuation shelter | | | | | | | | | | | Check the ones that the organization/profession was involved in (Check all that apply ☑） |
| Category | Menu | | Amount | Food groups （Check all that apply ☑） | | | | | Types of foods （Check all that apply ☑） | |  |
| Morning |  | |  | □Grain dishes (rice/bread/noodles) □Fish and Meat dishes (meat/fish/egg/soybeans) □Vegetable dishes (vegetables/mushrooms/potatoes/seaweed) (including vegetable juice, etc.） □Milk and dairy products □Fruit | | | | | □Hot meal service □Boxed meal □Food aid (ready-to-eat) □Stockpiled food (ready-to-eat) □Others： | | □Self-Defence Forces □Dietitian  □Others： □None of them were involved □Unknown |
| □Sufficient  □Insufficient  □Not provided  □Unknown |  |  |  |  |  |  |  |  |  |  |  |
| During the day |  | |  | □Grain dishes (rice/bread/noodles) □Fish and Meat dishes (meat/fish/egg/soybeans) □Vegetable dishes (vegetables/mushrooms/potatoes/seaweed) (including vegetable juice, etc.） □Milk and dairy products □Fruit | | | | | □Hot meal service □Boxed meal □Food aid (ready-to-eat) □Stockpiled food (ready-to-eat) □Others： | | □Self-Defence Forces □Dietitian  □Others： □None of them were involved □Unknown |
| □Sufficient  □Insufficient  □Not provided  □Unknown |  |  |  |  |  |  |  |  |  |  |  |
| During the night |  | |  | □Grain dishes (rice/bread/noodles) □Fish and Meat dishes (meat/fish/egg/soybeans) □Vegetable dishes (vegetables/mushrooms/potatoes/seaweed) (including vegetable juice, etc.） □Milk and dairy products □Fruit | | | | | □Hot meal service □Boxed meal □Food aid (ready-to-eat) □Stockpiled food (ready-to-eat) □Others： | | □Self-Defence Forces □Dietitian  □Others： □None of them were involved □Unknown |
| □Sufficient  □Insufficient  □Not provided  □Unknown |  |  |  |  |  |  |  |  |  |  |  |
| Snacks, sweets, alcohol, etc. |  | | | | | | | | | | |

**Supplementary material** Dietary Assessment Sheets for Evacuation Shelters

This is the first page of Dietary Assessment Sheet consisted of two pages. The second page includes living environment of the shelter and physical conditions of the evacuees.
